# Draft genome sequence of *Nocardia* anissiorum JW2 isolated from soil with high antimicrobial resistance to antimicrobial compounds secreted by *Streptomyces spp*

**DOI:** 10.1128/mra.00913-25

**Published:** 2025-10-29

**Authors:** Jaouad Anissi

**Affiliations:** 1 Euromed University of Fes (UEMF), Fez, Morocco; Montana State University, Bozeman, Montana, USA

**Keywords:** *Nocardia* sp., draft genome sequence, antimicrobial resistance

## Abstract

The draft genome of a multi-drug-resistant *Nocardia* anissiorum, designated JW2, isolated from soil, and exhibiting considerable resistance to anti-microbial agents produced by different *Streptomyces* species is provided. Genome mining counts for 35 biosynthetic gene clusters. A total of 37% of the identified BGCs have no similarity in the MiBIG database.

## ANNOUNCEMENT

*Nocardia* are Gram-positive bacteria of the phylum Actinobacteria, which are found in varied environments. Most of them are pathogenic for human or animal infections (1). Apart from their pathogenicity, *Nocardia* produce diverse antimicrobial metabolites produced from various biosynthetic gene clusters (BGCs) (2).

A bacterial isolate, *Nocardia* anissiorum JW2, was recovered from a barren land soil in the Middle Atlas (34° 3′45.76″N, 4°21′17.14″W), in Taza, Morocco. Approximately 0.5 g of soil at ~2 cm depth was used for isolation, based on standard protocols of re-suspension and serial dilutions. The bacterial growth and cultivation were done at 30°C for 4 to 7 d. All the steps of isolation and colony purification were done in Bennett medium.

**Fig 1:**
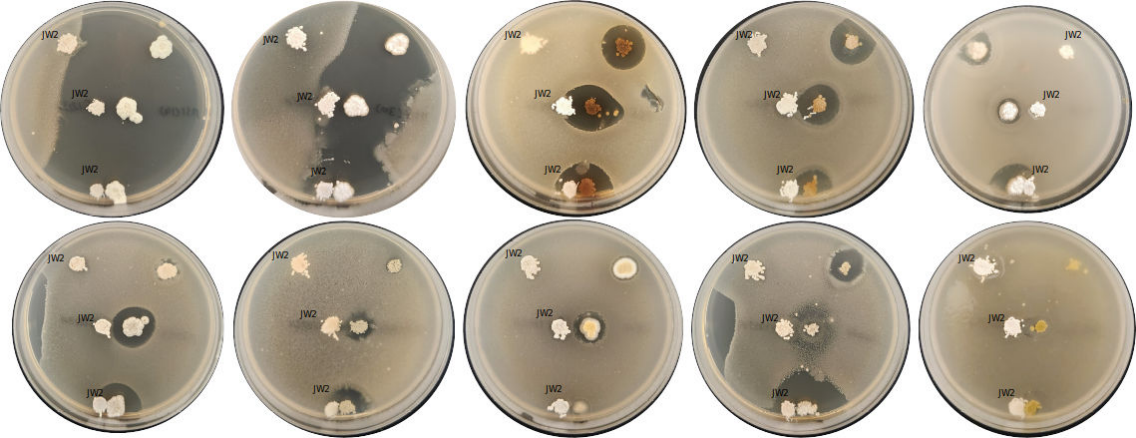
Typical examples showing the behavior of *Nocardia* anissiorum. JW2 in co-culture with other antimicrobial-producing *Streptomyces spp*. Co-culture was done on Bennett’s medium at 30°C for 4 days, and a layer of soft agar containing *Bacillus subtilis* ATCC 6051 was added to elucidate the secretion of antibacterial compounds in the medium.

*Nocardia anissiorum.* JW2 was selected along with 850 other strains (identified as *Streptomyces spp.,* on *the* basis of polyphasic taxonomy). These Actinobacteria were members of a “*national championship”* where Actinobacteria compete in a battle, as shown in the typical examples in Fig. 1. Strain JW2 showed resistance to almost all the antibacterial-producing *Streptomyces spp*.

gDNA was extracted from pure colony of JW2 grown in Bennett broth medium without glucose. After 5 days of incubation, gDNA was extracted using Monarch gDNA Purification kit, following the manufacturer’s instructions. The 16S rRNA fragment was amplified using One*Taq* from NEB and universal primers 27F (AGAGTTTGATCMTGGCTCAG) (3) and 1492R (GGTTACCTTGTTACGACTT) (4). Sanger sequencing using BigDye Terminator v3.1 Cycle Sequencing Kit and SeqStudio for sequence reading revealed that the 16S rRNA sequence of strain JW2 (PX113525) had 99.46% identity with *Nocardia ignorata* strain IMMIB R-1434 (NR_028006), 98.80% identity with *Nocardia fluminea* strain DSM 44489 (NR_114644), and 96.83% identity with *Nocardia camponoti* strain 1H-HV4 (NR_148835.1). However, based on CLSI MM18-A guidelines, this strain could not be identified as *Nocardia ignorata* (5).

Whole-genome sequencing was performed on Illumina platform NextSeq2000 generating paired-end reads of 151 bp. Library preparation was performed using Nextera XTT Sample Pre Kit Prep (Illumina, France). Raw reads were quality checked using FastQC v0.72 and trimmed using Trimmomatic v0.39 (6). *De novo* assembly was carried out using SPAdes v1.9 (7) with “--careful” flag and Shovill v1.1.0 (8). Assembly quality was assessed using QUAST v5.3.0 (9), which reported a total assembly length of 6.7 Mbp across 81 contigs, with an N_50_ of 251,732 bp and a largest contig of 836,211 bp. The average genome coverage was 21× and an average GC content of 68.08%. Genome annotation was done using Prokka (10), and BGCs were predicted using antiSMASH v7.1.0 (11). The antiSMASH analysis identified 35 BGCs, including 15 nonribosomal peptide synthetases (NRPSs), 7 polyketide synthases (PKS), 2 hybrid NRPS-PKS clusters, 4 terpenes, four RiPP-like compounds, and 13 other unknown BGCs. Default parameters were used except where otherwise noted.

## Data Availability

The genome assembly and raw reads have been deposited in GenBank under BioProject accession PRJNA1293937 and the assembly JBPXLK000000000.1. Reads were deposited under SRA accession SRR350285 (https://www.ncbi.nlm.nih.gov/sra?LinkName=biosample_sra&from_uid=50084355). The version of the data described in this paper is the first version.
